# Common immunopathogenesis of central nervous system diseases: the protein-homeostasis-system hypothesis

**DOI:** 10.1186/s13578-022-00920-5

**Published:** 2022-11-16

**Authors:** Kyung-Yil Lee

**Affiliations:** 1 The Catholic University of Korea College of Medicine, Seoul, Republic of Korea; 2 Junglock Biomedical Institute, 127 Yuchun-Ro, Jung-Gu, Deajeon, 34886 Republic of Korea

**Keywords:** Central nervous system diseases, Pathogenesis, Prion diseases, Alzheimer disease, Genetic diseases, Protein-homeostasis-system hypothesis

## Abstract

There are hundreds of central nervous system (CNS) diseases, but there are few diseases for which the etiology or pathogenesis is understood as well as those of other organ-specific diseases. Cells in the CNS are selectively protected from external and internal insults by the blood–brain barrier. Thus, the neuroimmune system, including microglia and immune proteins, might control external or internal insults that the adaptive immune system cannot control or mitigate. The pathologic findings differ by disease and show a state of inflammation that reflects the relationship between etiological or inflammation-inducing substances and corresponding immune reactions. Current immunological concepts about infectious diseases and infection-associated immune-mediated diseases, including those in the CNS, can only partly explain the pathophysiology of disease because they are based on the idea that host cell injury is caused by pathogens. Because every disease involves etiological or triggering substances for disease-onset, the protein-homeostasis-system (PHS) hypothesis proposes that the immune systems in the host control those substances according to the size and biochemical properties of the substances. In this article, I propose a common immunopathogenesis of CNS diseases, including prion diseases, Alzheimer’s disease, and genetic diseases, through the PHS hypothesis.

## Introduction

The central nervous system (CNS) controls the thinking, voluntary behavior, and involuntary movements of the vital organs, including the heart, lungs, and bowels, in all complex organisms. As shown by insights from evolutional, anatomical, and physiological research, the CNS might be the most important system in multicellular organisms, including humans. Scientists have studied hundreds of CNS diseases, but the etiology and pathogenesis of only a few of them are understood as well as those of other organ-specific diseases.

The human immune system consists of many kinds of immune cells, immune proteins, and other unidentified immune materials and might control toxic substances against the cells of various organs. The immune components of the host, including those involved in both the innate and adaptive immune systems, are seen in nearly all pathological lesions of infectious, autoimmune, and allergic diseases, cancers, wound healing, transplantation rejection, and organ-specific diseases, including CNS diseases. Because the nature of a disease at the molecular level involves the injury or malfunction of cells in specific organs, the pathological processes of the disease from initiation to recovery appear as pathohistological findings in the tissues of the affected organ. Thus, the pathologic findings from affected organ tissues at any stage of a disease can provide information about the battle situation between the immune system and inflammation-inducing substances. Likewise, the pathologic findings from CNS diseases provide information about disease processes and ongoing inflammation as the immune system works against etiological insults. It is possible that the immune cells, immune proteins, and undetermined immune materials, including amyloid proteins and peptides, that are observed in pathologic lesions might be associated with target-cell injury in CNS diseases.

All complex organisms are constructed of materials such as proteins, elements, and other biochemicals, and consist of cells, tissues, and organs. At the same time, living organisms are often regarded as unitary biological systems. Therefore, it is a philosophically baffling question whether any control system exists to maintain either cells or whole organisms in a healthy or homeostatic state. The host immune system reacts not only to pathogens but also to small substances that originate from pathogens, including toxins and pathogen-associated molecular patterns (PAMPs) [1], and substances originating from injured or infected host cells, including damage (danger)-associated molecular patterns (DAMPs), pathogenic proteins, and pathogenic peptides [2, 3]. Every disease involves etiological or inflammation-inducing substances, and current immunological concepts have limitations in explaining unresolved issues in the pathophysiology of disease. The protein-homeostasis-system (PHS) hypothesis proposes the existence of a system that controls all biological activity within organisms, including immune reactions; within the PHS, the immune system of the host is understood to control etiological substances according to their size and biochemical properties and to compensate for protein deficiencies in the host or within cells [4–7].

In this review, I use the PHS hypothesis to present a common immunopathogenesis of CNS diseases. Because the PHS hypothesis broadly integrates the pathogenesis of CNS diseases, concepts or details about the pathophysiology of specific CNS diseases that are important in the current immunological understanding are omitted or reinterpreted.

## Structure of the CNS and the blood–brain barrier

The cells in the CNS are protected by anatomical barriers that are specific to the CNS, such as the skull, meninges, and blood–brain barrier (BBB). The CNS contains a variety of anatomical structures, including the brain, spinal cord, and other specialized parts, each of which can consist of different nerve cells and supporting cells that perform specific roles to maintain the CNS in a healthy state. For example, specialized neurons and supporting stromal cells have different receptors for affinitive materials and produce different proteins and biochemicals, as well as cells in a variety of organs. At the cellular level, the receptor binding-signal transduction mechanisms are established as a basic mechanism of life in all organisms. Thus, a substance toxic to certain organ cells is not necessarily toxic to other organ cells that lack affinitive receptors. Because each cell can have its own receptors responsible for binding toxic substances, inflammation begins when substance–receptor binding pathways are activated, with beginning a cascade of corresponding immune reactions against the substances [5]. Most neurons in the CNS primarily express receptors for monoamines and neurotransmitters, and they sparsely express pattern recognizing receptors (PRRs) and receptors for immune proteins such as cytokines and chemokines [8]. However, major histocompatibility complex (MHC) class I molecules, which might be a self-identifying cell marker for immune reaction and brain development, are expressed in most neurons [5, 9]. Cells in the CNS are selectively protected from external and internal insults by the BBB, which consists of endothelial cells and glial cells and acts as a wall of protection against external invaders such as bacteria, viruses, fungi, and parasites. Furthermore, the BBB does not permit most serum proteins or peptides or external materials such as chemicals (drugs) or biochemicals to pass [10]. However, BBB structures are lacking in several anatomical regions, including the roof of the third and fourth ventricles, the roof of the diencephalon, and the pineal gland. The pineal gland secretes the hormone melatonin directly into the systemic circulation [11]. Although the reasons for the existence of those BBB-deficient structures remains to be elucidated, recent studies have suggested that the CNS immune system can communicate with the systemic immune systems via vascular channels [12, 13]. It is possible that there are gates that can be opened immediately when extensive immune cells and immune proteins are needed, such as in the case of acute bacterial meningitis.

Cerebrospinal fluid (CSF) is produced by epithelial cells in choroid plexus and looks clear, but it contains various monoamines, neurotransmitters, electrolytes, and a small part of serum proteins (0.3%) and immune cells, mainly T cells, in healthy persons [14, 15]. CSF profiles, such as cell counts, protein and lactate levels, the CSF/plasma albumin ratio, and culture and polymerase chain reaction assays are used to diagnose CNS infections and inflammatory conditions. The components of CSF have been extensively evaluated as biomarkers for various neurological disorders, including infections, inflammatory or neurodegenerative disorders, seizure disorders, tumors, traumatic brain injury, and hydrocephalus [16–18]. The biomarkers of each neurologic disease are helpful for diagnosing and evaluating disease processes and include proteins (such as immunoglobulins or the oligoclonal band, tau protein, glial fibrillary acidic protein, neuron-specific enolase, neopterin, S100β, and interleukins) and other small components such as micro RNAs, mitochondrial DNA, reactive oxygen species, and changed level of monoamine metabolites in children and adults [16–18]. Because cells in the CNS communicate through the CSF, most biomarkers, including immune-associated proteins and DAMPs, are associated with activated neuroimmune cells, such as microglia and astrocytes, and injured target CNS cells. Thus, certain proteins in the CSF could play roles in the immune reactions to CNS disorders.

## Immune systems in CNS

Multicellular organisms, including human beings, have evolved from unicellular organisms such as bacteria and yeast, which possess an innate immune system in the form of proteins such as restriction enzymes that protect against external insults such as bacteriophage infection and natural toxins [19]. Plants and invertebrates, including insects, evolved far earlier than mammals and still have only innate immune systems. In insects, the major immune mechanism consists of phagocytes with Toll receptors and immune protein systems, including complements and antimicrobial peptides such as defensins [20]. Among the descendants of ancient eukaryotes, only jawed vertebrates, including humans, have an adaptive immune system alongside their innate immune system. Thus, it is a fundamental assumption that the immune systems of each organism have evolved to protect their cells.

Current immunological concepts have developed and been established mainly through studies of invading pathogens. Although the scientific community has divided the immune system into two major subsystems, innate and adaptive, all immune cells and immune proteins in both systems perform their functions during noninfectious events, such as trauma (or wound healing), transplantation rejection, allergy, intoxication, and cancer. In addition to an invasion of external pathogens, some microbial species in the microbiota can invade the host. In fact, most currently problematic infectious diseases are caused by strains derived from the microbiota of human beings; they far exceed diseases caused by external pathogens from animal species or some other origin. Thus, it is possible that the adaptive immune system has mainly evolved to protect against internal insults, such as toxic substances derived from injured or infected self-cells, as well as insults caused by an invasion of dysbiotic strains in the microbiota or external pathogens. Accordingly, there might be mechanisms for reducing the substances derived from self-cells; apoptosis, autophagy, DNA traps laid by immune cells, and epigenetic changes such as micro-RNAs and gene methylation within cells might be the players that perform that critical role at least in part [5]. Because the neuroimmune system of mammals is separated from the systemic immune system by the BBB, blood–CSF barrier, and similar fluid–brain barriers, the CNS contains few circulatory antibodies and T cells at the steady state. On the other hand, peripheral- and bone marrow-derived immune cells such as macrophages and T cells reside in the choroid plexuses in stroma of the ventricles, CSF, and meninges, suggesting that the neuroimmune and systemic immune systems might communicate with each other [12, 13, 21].

In the neuroimmune system, microglia, astrocytes, and mast cells (MCs) are the major immune cells [22, 23]. Microglia are the resident innate-immune cells most abundant in the CNS, and they perform basic innate immune functions such as phagocytosis, antigen presentation, and the activation of inflammatory responses against DAMPs and PAMPs. The microglia have a variety of subtypes and express most of the Toll-like receptors (TLRs), whereas the astrocytes and neurons express only a limited set of PRRs. Also, the microglia play critical roles during neurodevelopment, including neuronal development and differentiation, through the production of cytokines and chemokines [24, 25]. Astrocytes are the most abundant glial cells in the brain and might be derived from neural progenitor cells. Although astrocytes are not considered to be part of the neuroimmune system, they express the PRRs for detection of PAMPs and MHC II molecules for antigen presentation, and they modulate the neuroinflammatory response through cytokine production [26]. Astrocytes have a diverse array of functions, including the maintenance of BBB integrity, cerebral blood flow, neurotransmitter metabolism, and nutritional support. Additionally, they communicate with adjacent glial, neuronal, vascular, and immune cells to regulate neural excitability and synapse formation [27, 28].

MCs are one of important effectors of the immune system and are well-known for their role in allergic diseases and anaphylaxis [29]. Also, MCs play important protective roles in wound healing, angiogenesis, immune tolerance, pathogen defense, and BBB function [30]. MCs are in all vascularized tissues, including the CNS, but dominantly reside at environmental interfaces, such as the skin, lungs, and intestinal tract. In the CNS, MCs reside in the pineal gland, neurohypophysis, hippocampus, and thalamus, and they are usually juxtaposed with the BBB vasculature and often closely associated with neurons and glia cells [23]. MCs have heterogeneous clonal subtypes in each tissue, but their main function might be the protection of self-cells as part of the innate immune system. MCs are activated by insults from infections, including COVID-19, and in other conditions eliciting allergic reactions and anaphylaxis [29, 31]. MC activation promotes the release or synthesis of hundreds of multi-action mediators, including biogenic amines (e.g., histamine), proteases (e.g., tryptase and chymase), cytokines (e.g., interleukins and tumor necrosis factor-α (TNF-α)), eicosanoids (e.g., prostaglandins and leukotrienes), heparin, and growth factors [23]. Although the etiological substances required for MC activation can differ in various conditions, at least some mediators derived from activated MCs could play key roles in eliciting the clinical manifestation of disease. Traditionally, systemic mastocytosis or systemic mast cell activation disease is known as a rare neoplastic disease characterized by mast cell infiltration in various tissues including skin and bone marrow. Patients with systemic mastocytosis show overt symptoms such as flushing, pruritis, and higher risk of anaphylaxis, with increased levels of tryptase, histamine, and other mast cell mediators [32]. On the other hand, MC activation syndrome (MCAS) was recently recognized. It is similar to systemic mystocytosis, but it has little to no clonal expansion and is far more prevalent than systemic mastocytosis. Patients with MCAS exhibit a wide range of acute or chronic illnesses that are characterized as multisystem, polymorbid inflammatory and/or allergic themes with wide arrays of neurologic and psychiatric symptoms. Conversely, a part of patients with neurologic and/or psychiatric symptoms who are caused by activated MCs in CNS could be accompanied with by other symptoms of MCAS [33]. The substances causing MC activation or mediators from activated MCs in the periphery could be transported via the bloodstream to the brain, where their effects are integrated with MC-mediator-related immune responses in CNS. All cells in organisms can communicate with one another through networks of the circulatory, lymphatic, and nervous systems, including the peripheral nervous system, endocrine pathways, and possibly an MC-associated network. Furthermore, neurons might communicate directly with immune cells such as microglia, astrocytes, and MCs in an integrated control system or network of cells that acts against disordered insults [34, 35]. It is possible that dysregulation of MC activation in the CNS is associated with both neuropsychiatric and allergic diseases, including intrinsic asthma and atopic dermatitis, because an acute emotional upset can induce an immediate asthma attack or aggravate of itching in atopic dermatitis.

Although the innate immune system in mammals, including granulocytes, monocytes, and MCs, has no specific (or adaptive) immune function against antigens, it has been proposed that innate immune system in mammals as well as in plants and insects has an inheritance of acquired immune resistance, named trained immunity, which may be associated with epigenetic reprogramming of transcriptional pathways rather than gene recombination [36]. The progeny of previously infected mammals has a memory of infection that confers enhanced protection against infection that is like that provided by the adaptive immune system [37]. During a disease insult, diverse epigenetic factors, including non-cording RNAs (long non-cording RNAs and microRNAs) and gene methylation, are activated, and the components or effectors of those epigenetic systems also work to protect self-cells against disease insults. Briefly, the effectors in epigenetic systems control genes, and the genes produce mainly proteins that are needed against insults, including immune-regulatory proteins for innate and adaptive immune cells. In other words, disease processes are controlled by proteins whose production is determined by genetic and epigenetic factors, which implies the existence of an integrated protein control system in the host. Thus, it is possible that the dysregulation of systemic protein homeostasis contributes to the pathogenesis and phenotypic characteristics of disease, including neurodegenerative diseases.

Inflammation and neuronal injury in CNS diseases might begin with binding between toxic or signaling substances and the receptors of the target cells, and those substances could originate from pathogens or from cells affected by infection, trauma, or other conditions. Because cells in the CNS mainly express receptors for biochemicals such as monoamines and neuropeptides, it is possible that substances toxic to neurons have similar size and biochemical properties to those receptors. The CNS immune system should thus control both toxic substances and similar substances secondarily released by injured or otherwise compromised CNS cells.

## Protein homeostasis (proteostasis) and the proteosome

All human cells have a genome that can produce any protein, but cells in different organs produce different proteins and express different receptors, as determined by the cell fate [38]. Each cell in a multicellular organism can have a structure and function similar to those in unicellular eukaryote organisms, which is why some physiological functions of human cells, such as autophagy, can be established through studies of unicellular organisms, such as yeast [39].

Protein homeostasis (proteostasis) is generally held to be important for maintaining cellular metabolic processes and survival. Within cells, proteins are produced and undergo a process of folding for activated forms and degradation of them as needed. The mechanisms for protein folding, conformational changes, and degradation through the ubiquitin–proteasome system (UPS) and autophagy-lysosomal pathway have been well described. It is generally understood that proteasomes degrade unneeded, misfolded, or damaged proteins by proteolysis and regulate the concentration of proteins using ubiquitin, ubiquitin ligases, chaperones, and co-chaperones. [40]. Growing evidence suggests that dysregulation of the UPS or autophagy is related to diseases including several neurodegenerative, infectious, and inflammatory disorders, and cancers [41].

Proteasomes are protein complexes inside the nucleus and cytoplasm of all eukaryotes, archaea, and some bacteria. Multiple proteasomes are present in a single cell type, and there is a variation between proteosome types and tissue cells, suggesting that the proteasome is optimized for each cell’s function with generation of distinct sets of peptides [42]. The proteasomes are found in the blood or are activated in peripheral immune cells of patients with multiple myeloma or infectious diseases, including COVID-19, suggesting that they might be involved in the pathophysiology of disease [43]. On the other hand, during protein degradation, variable sized peptides of 5–22 amino acids long are produced, and some of them are biologically active and can attach to cellular receptors [44]. Proteasome inhibitors, including bortezomib (Velcade), and inhibitors of enzymes linked to UPS, including thalidomide and lenalidomide, are effectively used to treat multiple myeloma and some hematologic malignancies and immune-mediated diseases [41, 45]. The mechanisms of proteasome inhibitors remain elusive, but proteasome inhibitors may inhibit protein degradation by blocking the processes of central canal of the relevant proteasomes and decrease a level of intracellular peptides markedly [45]. Although the drugs are thought to induce apoptosis by disrupting the regulated degradation of proteins involved in the pro-growth cell cycle, their anti-tumor or apoptosis-inducing effect appears selectively across tissue cells, even though all cells, including tumor cells, contain proteosomes [42, 46]. It has been suggested that the disturbance of proteostasis is associated with some neurodegenerative diseases, including Alzheimer disease (AD), Parkinson’s disease (PD), Huntington’s disease (HD), and amyotrophic lateral sclerosis based on the pathological observations that aggregated peptides or proteins are found within cells in these diseases. Also, therapeutic strategies suggested for those diseases are based on the concepts of proteostasis [47, 48].

Because the cells of multicellular organisms communicate with each other via networks, most proteins produced within a particular cell group are not used by those cells but by other cells as required by or for communication across cells and systemic homeostasis of the organism. Furthermore, not only proteins but also peptides; neuropeptides; monoamines; elements such as NO, and O_2_; and biochemical substances such as vitamins and fatty acids all have crucial roles to ensure the homeostasis of the organism and its cells. These smaller substances also have receptors on or in specific target cells and specialized signal-transduction pathways. Because a cell can reasonably be regarded as an independent biosystem, cells also have protective mechanisms against external insults (such as pathogen invasion) or physical stress (such as heat or trauma); for example, virus-infected cells produce interferons and activate proteins associated with interferon-related pathways, and heat-stressed cells activate proteins, including heat shock proteins, within their proteostasis networks.

On the other hand, because a cell is too small to store all the proteins required to maintain homeostasis, each cell contains only the essential proteins that are needed for survival or for maintaining its own homeostasis. Given that similar peptidomes and proteosomes exist in cells of all organisms including unicellular organisms [44, 49], it is possible that smaller substances, such as peptides, might be the main effectors of biological tasks in the normal or steady state cell environment. Also, it is reported that peptides for T cell receptor (TCR) recognition of T cell clones and MHC class I are produced in proteosomes in antigen present cells [50]. Because peptides are not encoded in the genome but nonetheless play crucial roles in organisms, proteosomes might be manufactured to produce the peptides needed for cellular homeostasis. In other words, blocking the production of essential peptides using proteosome inhibitors might induce the apoptosis or malfunction of certain cells, including multiple myeloma cells, by depriving them of essential peptides. Also, cells affected by trauma, infection, or other insults contain numerous peptides that can induce inflammation if they are released and bind to target cells.

## CNS diseases and the microbiota

All organisms appear to have co-evolved with microbiota in each species. Microbiota consists of various strains of bacteria, viruses, and fungi that reside in the host’s oropharynx and lower respiratory tract, gastrointestinal tract, skin, and urogenital tract. Strains in the microbiota have a collaborative partnership with the host, including providing nutritional or vital materials and preventing colonization by external pathogens. Furthermore, the mucosal immune system, such as gut-associated lymphoid tissue, is established after colonization by normal gut flora, and the resulting gut microbiota influence the neuroimmune system by affecting, for example, microglial maturation and function [51, 52]. Germ-free or antibiotic-treated mice have high numbers of immature microglia and express different genes with impaired induction of proinflammatory cytokines such as interleukin (IL)-1β, IL-6, and TNF-α, compared with conventionally colonized controls [53]. Those findings suggest that a homeostatic state between a host and their symbiotic microflora is critical for maintaining the well-being of the host and that the microbiota and the host immune systems are closely linked.

The strains in microbiota within individuals are changing continuously after birth together with immune maturation [54]. Also, the component strains of microbiota in different ethnic groups are influenced by environmental factors such as diets and socioeconomic factors such as antibiotic use [55]. Thus, the microbiota can differ in various populations and can be changed by a changing environment. The disruption of reciprocal equilibrium between the microbiota and the host, dysbiosis, has been reported in various diseases, including obesity, autoimmune diseases, and cancers [56, 57]. Researchers have also reported on the relationships between intestinal microbiota and neuropsychiatric diseases through hypothesizing a microbiota–gut–brain axis [58]. Dysbiosis of the gut microbiota with a dysregulated immune response, including microglial activity, have been studied in neuropsychiatric disorders, including multiple sclerosis, amyotrophic lateral sclerosis, PD, stroke, autism spectrum disorder, anxiety, and depression [59]. The mechanisms by which dysbiosis provokes those diseases remain elusive. The overproduction of toxic materials by dysbiotic microbials, vulnerability to invasion by microbials through weakened mucosal barriers, and disruption of the homeostatic relationship between microbiota and the host’s immune system, including microglial development and function, have all been suggested to be associated with disease onset [60, 61].

Susceptibility to disease, especially immune-mediated disease, has been widely explained using genetic or environmental factors. The prevalence or incidence of infection-related immune-mediated diseases in childhood, such as Kawasaki disease (KD), multisystem inflammatory syndrome in childhood (MIS-C), juvenile idiopathic arthritis (JIA), type I diabetes, inflammatory bowel disease, and Bechet disease, differs quite widely among different populations [4, 7]. For example, the incidence of KD is 10–30 times higher in East Asian countries such as Japan and South Korea than that in European countries, whereas that of JIA is more than 10 times higher in Northern European countries than in East Asian countries [62]. In addition, some immune-mediated diseases, such as KD and subgroups of JIA, have a strict predilection of age or sex. The strains in microbiota can cause infectious events and postinfectious immune-mediated diseases, and those strains can first colonize and then invade the host. Thus, the different incidences of disease among ethnic groups or populations and the vulnerability associated with age or sex can be explained by colonization state of etiological agents in the microbiota [4, 7, 63]. The clinical manifestations and immune functions of patients with KD, JIA, and infection-related CNS diseases, including transmissible spongiform encephalopathies (TSEs), are similar across populations. Accordingly, it is possible that patients or animals with infection-related CNS diseases such as kuru or animal TSEs have a high chance of being exposed to pathogens that originate from microbiota, similar to patients with KD or JIA who live in areas with a high prevalence of those conditions [4, 7, 63].

## PHS hypothesis for CNS diseases

Every disease has etiologic substances that are associated with disease-onset or the initiation of inflammation, although most of them have yet to be identified. The etiological or triggering substances associated with disease onset are small materials, including elements, biochemicals such as monoamine metabolites, chemicals (drugs), and pathogenic proteins and peptides. Those small substances can be divided into 2 groups, proteins and non-proteins by biochemical characteristics. In infectious or infection-related immune-mediated diseases, including influenza, COVID-19, KD, and MIS-C, the viruses or bacteria themselves are not directly toxic to the host cells. Instead, small substances produced as the result of infection, such as toxins, PAMPs, DAMPs, and other materials derived from infected cells (the focus), could be responsible for target-cell injury [7, 64, 65].

In addition to known DAMPs and PAMPs, numerous unidentified inflammation-inducing substances exist within host cells. Clinical observations have suggested that substances from certain organ-specific cells can affect other cells in the same or different organs. For example, severe pneumonia and acute respiratory distress syndrome can be caused by nonpathogen-associated insults such as blunt chest contusion, gastric content aspiration, multiple injuries, pancreatitis, burns, inhalation of toxic gas, and amniotic fluid embolism [65]. Also, abrupt cerebral edema caused by a cerebral contusion could be related to substances derived from cells injured by trauma. Furthermore, intracellular organelles, including mitochondria, can secrete various components into the extracellular circulatory system, including mitochondrial DNA, formylated peptides, and ATP, when cells are injured [66]. Researchers have already reported a relationship between various DAMPs and chronic neurologic diseases including AD [68].

The PHS hypothesis has been introduced as a new interpretation to shed light on previously unresolved immunological issues in various diseases [4–7, 64, 65, 67]. In brief, a living organism is a biological system, and all the biological activities, including embryogenesis, immune reactions, and adaptation to protein deficiencies, are controlled by an integrated system named the PHS. The adaptive immune system controls pathogenic or etiological protein substances; B cells control pathogenic proteins through antibody production, and T cells control pathogenic peptides being sized for TCR binding, possibly through TCR-related immune reactions. T cells and B cells use a recombination of immune genes that code for TCRs and B cell receptors to control a variety of pathogenic proteins and peptides, respectively. In the innate immune system, phagocytes control larger substances, such as whole viruses, bacteria, and apoptotic or necrotic bodies, by means of phagocytosis, and TLR-associated immune reactions, natural antibodies, complements, and other immune protein systems control smaller, non-protein, toxic substances such as lipopolysaccharides, viral DNAs and RNAs, biochemicals, and small peptides that cannot be controlled by T cells.

**Fig. 1:**
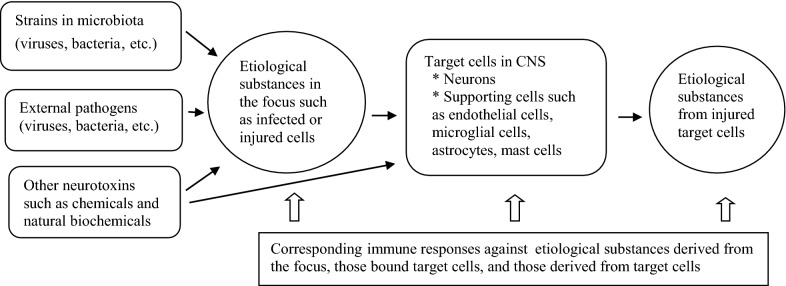
Schematic diagram of immunopathogenesis of CNS diseases

The injury of target cells in autoimmune diseases might not be caused by specific antibodies or T cell clones against antigens expressed on self-cells but rather by persistent aberrant immune reactions between nonspecific adaptive immune cells and substances produced by injured self-cells. Thus, patients with autoimmune diseases have an improper immune status, especially adaptive immune–cell clones, against pathogenic proteins or pathogenic peptides derived from injured cells. Certain CNS diseases, including prion diseases and AD, and amyloidosis-related disorders, involve the infiltration of amyloid proteins without adaptive immune reactions involving immunoglobulins or lymphocytes at the initial pathologic lesions. Because under the PHS hypothesis, early pathologic lesions are presumed to involve etiological substances and corresponding immune reactions, the etiological substances in disease must be very small and controlled by the innate immune system, which is composed of immune proteins or peptides that are undefined at this time. The etiology of genetic diseases and cancers could involve a transformed protein or protein deficiency in organ tissue or within a cell. Adapted reactions such as the production of an alternative or compensatory protein and the subsequent disruption of protein homeostasis would then be responsible for disease development and progression through long-term, nonspecific, hyperactive reactions associated with those proteins [6] (Table 1 and Fig. 1).

**Table 1:** Etiological substances and corresponding immune effectors in the PHS hypothesis

Etiological substances (or events)	Corresponding immune effectors
Pathogenic proteins (BCR-associated)	B cells: antibodies against pathogenic proteins
Pathogenic peptides (TCR-associated)	T cells: peptides or cytokines against pathogenic peptides
Pathogenic small peptides, monoamines, their metabolites (especially in CNS)	Immune proteins such as PrP gene products and other amyloid proteins, mast cell-associated immune responses
Non-protein materials such as LPS, RNAs, DNAs, chemicals, biochemicals	TLR-associated immune responses, natural antibodies, other immune systems such as complements and other proteins
Large complex substances such as viruses, bacteria, parasite, apoptotic & necrotic bodies	Phagocytes (neutrophils and macrophages), eosinophils (in case of large parasites)
A protein deficiency or malfunctioning protein in organ tissues or within a cell	Production of alternative proteins in genetic diseases and cancers

*BCR* B cell receptor; *TCR* T cell receptor; *PrP* prion protein; *CNS* central nervous system; *LPS* lipopolysaccharide; *TLR* Toll-like receptor

## Infectious diseases and infection-related immune-mediated diseases of the CNS

Sometimes a variety of pathogens, such as viruses, bacteria, fungi, and parasites, can invade the CNS. The pathophysiology and mechanisms of the host immune system in reaction to infectious CNS diseases and infection-related immune-mediated diseases could be same as those associated with infectious diseases in other bodily regions. Every infectious disease has the focus which has etiological substances involved in disease-onset and contains replicated pathogens, byproducts from pathogen replication processes (such as toxins, viral DNAs and RNAs, and PAMPs), or substances derived from injured cells (such as DAMPs, pathogenic proteins, and pathogenic peptides). On occasion, those substances can spread via systemic or local circulation, bind to target cells, and begin to induce inflammation. Simultaneously, corresponding immune reactions are activated in a variety of immune substances, including cytokines and proteolytic proteins. Moreover, it has been suggested that hyperimmune or aberrant immune reactions, such as cytokine storms, could be related to target cell injury [7, 65]. It is reasonable to think that the severity of infectious and infection-related diseases, including those in the CNS, depends on the amount of etiological substance produced and the corresponding immune reactions. Because inflammation-inducing substances and the substances responsible for target cell injury might be not pathogens themselves, it is unsurprising that pathogens cannot be detected in CSF or the pathologic lesions of most patients, especially those with intracellular infectious diseases caused by viruses or mycoplasmas [64, 65].

The clinical manifestations and prognosis of postinfectious immune-mediated diseases in the CNS, including postinfectious encephalopathy, Guillain–Barre syndrome (GBS), acute disseminated encephalomyelitis (ADEM), and other acute and chronic neurological diseases, differ in individual patients. In infectious diseases such as meningitis and brain abscesses or infection-related immune mediated diseases such as GBS and ADEM, intact bacteria and viruses could be controlled by phagocytes such as neutrophils and macrophages in the blood and possibly by localized microglia, while smaller substances such as pathogenic proteins and peptides and other biochemicals would be controlled by corresponding immune components in the innate and adaptive immune systems. Therefore, immune cells and immune proteins might enter the CSF and pathologic lesions through the free zone of the BBB or other gates, not through a broken BBB. It has also been proposed that if the immune system of the host cannot control toxic substances derived from initially infected cells or target cells, ongoing target cell injury caused by persistent activation of a nonspecific adaptive immune reaction to those substances will cause the development of chronic inflammatory diseases or autoimmune diseases [5–7].

Acute encephalopathy can occur during or after acute infections such as influenza and COVID-19 [69, 70]. As a specific type of acute encephalopathy, Reye syndrome occurs in some children who take aspirin during acute viral infections such as influenza or chicken pox. Reye syndrome was once prevalent in the era of common use of aspirin and is characterized by vomiting, tachypnea, and coma that could be caused by acute cerebral edema and transient liver cell dysfunction [71]. The pathologic findings show generalized edema of neurons without destructive lesions and no infiltration of immune cells or immune proteins such as immunoglobulins and complements. Also, the affected liver cells show generalized fatty degeneration without liver cell damage or the infiltration of immune cells or antibodies. The pathogenesis of Reye syndrome remains unknown, though mitochondrial dysfunction and toxic substances associated with aspirin have been suggested [72]. It is possible that the etiological substances in Reye syndrome are produced by certain pathogen-infected cells that are influenced by aspirin (as the initial focus) and have an affinity for the receptors on neurons or other CNS cells, and liver cells. Those substances can easily penetrate the BBB, bind to the receptors of target cells in the CNS, and activate corresponding immune response and receptor-associated pathways. During those immune processes, excessive amounts of the substances would induce an extensive reaction of immune products derived from CNS immune cells, i.e., microglia, possibly astrocytes or MCs, and what was initially a hyperactive nonspecific immune reaction against the etiological substance could become responsible for acute generalized cerebral edema. These mechanisms could also apply to other types of pathogen-associated acute encephalopathies that show similar pathologic findings. Thus, an excess of an etiologic substance that cannot be controlled by the innate immune system of the CNS can induce severe cerebral edema and even death, as shown by similar phenomena in cases of cytokine storms caused by severe systemic immune reactions to large amounts of etiological substances [7, 65].

The pathogenesis of postinfectious diseases has previously been explained by 2 main mechanisms: direct injury caused by pathogens, or an immune mechanism caused by specific antibodies or specific T cell clones based on the molecular mimicry hypothesis. Most postinfectious immune-mediated diseases in the CNS, including acute encephalopathy, GBS, and ADEM, appear 1–4 weeks after clinical symptoms of the initial infection have subsided. In those diseases, it is difficult to detect etiologic pathogens or components of pathogens in the CSF or pathologic lesions, as with other organ-specific postinfectious immune-mediated diseases, suggesting that the toxic or inflammation-inducing substances might be derived from infected or injured self-cells, including neurons.

A CNS disease entity, such as an autoantibody-related CNS disease or autoimmune CNS disease, has been suggested [73, 74]. The autoantibodies, including anti-NMDAR antibody and anti-GQ1b antibody, may be produced against protein antigens from injured neuronal cells and detected in the blood and/or CSF on occasion. Although autoantibodies are believed to be associated with pathogenesis of diseases, in the PHS hypothesis, specific antibodies or T cell clones to substances derived from self-cells are not the cause of disease; instead, they represent the preventive immune response to possibly toxic substances derived from self-cells [5]. Antibodies that react to components within self-cells, so-called autoantibodies, are commonly observed in immune-mediated diseases, including systemic lupus erythematosus (SLE) and JIA, as well as in autoantibody-related CNS diseases. On the other hand, some autoantibodies exist in healthy persons, patients with cancers such as teratoma, and patients in complete remission. Moreover, some kinds of autoantibodies are detected in clinically diverse immune-mediated diseases, and multiple autoantibodies can appear in a single autoimmune disease entity.

Immune modulators such as corticosteroids and intravenous immunoglobulin (IVIG) have been used to treat CNS diseases, including autoantibody-related CNS diseases, but their effects differ depending on the disease entity and even in identical disease phenotypes. In the PHS hypothesis, the appearance of specific antibodies or T cell clones in blood or pathologic lesions indicates that they are reacting to pathogenic proteins or peptides, including those derived from injured cells. Thus, a high level of specific autoantibodies could be related to disease severity by indicating a high number of injured host cells that warrants a large active immune reaction in response to high amounts of the etiological substance. If the pathophysiology of a certain CNS disease is associated with acute adaptive immune system activation, as is found in most immune-mediated and autoimmune diseases, including GBS, ADEM, SLE and vasculitis syndrome, and in infectious diseases, including viral pneumonia caused by cytokine storms, early properly dosed corticosteroids and/or IVIG based on clinical severity of the patients could effectively reduce the morbidity of the disease [7, 64, 65, 67]. However, those drugs might be ineffective or take a long time to be effective in CNS diseases whose pathophysiology is associated with the innate immune system, such as the immune-protein system. As previously proposed, patients with autoimmune diseases, including CNS diseases, have improper immune status for controlling initial exposure to an etiological substance to protect against target cell injury or control toxic substances produced by injured target self-cells. The immune system, especially the adaptive immune system, matures with age until adulthood and then declines in old age [75]. Therefore, the clinical features and prognosis of immune-mediated CNS diseases associated with the adaptive immune system could differ between children and adults. Children with autoantibody-related CNS diseases, especially young children, could have a better prognosis as their adaptive immune function matures over time, as is found in some immune-mediated diseases such as JIA, infantile eczema, and childhood idiopathic thrombocytopenic purpura [4, 65]. Because immune modulators induce only early stabilization of hyperimmune reactions, they cannot control etiological substances or cure disease.

## TSEs (or prion diseases), AD, and amyloidosis

Because a disease state begins when etiological substances bind to affinitive receptors on target cells, the innate immune system of immune proteins or immune peptides might control the smallest biochemical substances. Thus, this concept of the innate immune system could provide new solutions to unresolved issues in the pathophysiology of subacute or chronic CNS diseases, including TSEs, AD, and PD. Pathologic findings common across those diseases are neuronal necrolysis, gliosis, and the infiltration of amyloids from outside the affected cells without the presence of adaptive immune components such as T cells or antibodies [76]. Clinical manifestation and the degree of a pathologic finding such as spongiform degeneration are quite variable between individuals, and the extent of reactive gliosis correlates with the degree of neuron loss, suggesting that disease progression is controlled by the immune system of the host.

TSEs or prion diseases are subacute or chronic degenerative CNS diseases. Human TSEs include the classic and variant forms of Creutzfeldt-Jakob disease (CJD), Gerstmann-Sträussler-Scheinker syndrome (GSS), fatal familial insomnia (FFI), and kuru, and animal TSEs include scrapie in sheep, bovine spongiform encephalopathy (BSE), and chronic wasting disease (CWD) in deer and elk. Although the etiopathogenesis of TSEs remains to be further evaluated, the prion theory suggests that pathogenic proteins, that is, prions, are responsible for disease-onset and transmission [77]. Also, familial CJD, FFI, and GSS are genetically determined by mutations of the PrP gene. Prions (PrP^Sc^) are isoforms of the product of normal PrP gene (PrP^C^) in mammalian cells, and the amino acid sequences of PrP^Sc^ and PrP^C^ are identical. PrP gene is a small, single-copy, housekeeping gene on chromosome 20 and the entire open reading frame is in one exon [78]. The biological function of PrP^C^ is unknown, and PrP^C^ can be transformed to different isoforms in vitro, but some PrP^Sc^ produced in vitro lack infectivity [79]. Proteins with self-propagating conformations have been found in single-cell organisms such as fungi and bacteria [80, 81], suggesting that conformational changes of certain proteins to protect against certain insults might occur commonly in the biological world. Many studies have supported prion theory. Transgenic mice that receive mutant PrP gene transplants can be affected with TSEs spontaneously, and brain extracts from patients with genetic prion diseases, including familial CJD or FFI, can experimentally transmit those diseases to animals [82]. Furthermore, PrP gene knockout mice are protected from TSE onset [83].

On the other hand, scrapie in sheep or animal model of scrapie (mice or hamsters) shows species-specificity, as shown in other infectious diseases including influenza, coronavirus or mycoplasma infections; susceptibility to the disease is associated with an individual’s genotype or with strains in a single animal species [84]. Also, historically, the avoidance of inbreeding in sheep halted scrapie outbreaks in the middle of the eighteenth century [85]. These findings suggest that genetic traits in the host immune system, including the PrP gene, are associated with disease susceptibility and the progression caused by infectious insults. Patients with classic CJD or kuru are sporadically affected, and most of them have a normal PrP gene with possibly conversion of PrP^C^ into PrP^Sc^ on occasion. In genetic diseases of PrP gene variants such familial CJD, GSS, and FFI, clinical neurologic characteristics and the involved pathologic lesions differ by disease. Moreover, the neurologic symptoms appear in middle age, and the clinical course differs between individual patients in the same family. The genetic form of these diseases is not contagious to other persons, though it can be transmitted to animals experimentally. These findings suggest that PrP^Sc^ is not a direct causative toxin or agent of disease onset. Instead, the products of a dysfunctional PrP gene might be associated with disease progression as an effector of immune regulation. Under the PHS hypothesis, the etiological substances of TSEs are smaller than prions. Thus, it is possible that the materials used in prion transmission studies, such as supernatants of animal brain tissues and isolated prion samples, contain smaller substances, such as oligonucleotides of fewer than 50 bases or even smaller biochemicals, along with prions [86]. It is also possible that some of those substances might be neurotoxins and initiate the nerve cell injury that sparks the corresponding immune reaction. Furthermore, the etiological substances in TSEs might be associated with an infection of pathogens, including dysbiotic strains of species-specific microbiota caused by changed diets, because certain TESs such as kuru and BSE, are associated with prolonged ingestion of human dead or the processed remains of other cattle, respectively. The incidence of CWD in cervids living in the wild has been estimated to be as high as 15%, whereas in captive populations, up to 90% of mule deer have been reported to be positive for prions [87, 88]. Those findings suggest that only some infected animals are affected by symptomatic CWD and that environmental factors such as feeding food are associated with prion infection. In neuronal cell lines, infection with the neurotropic influenza A virus induced the conversion of PrP^C^ into PrP^Sc^ and the formation of infectious prions [89], and mice co-infected with prions and murine adenovirus showed accelerated prion disease [90], suggesting that the conversion of PrP^C^ into PrP^Sc^ is associated with infectious insults to cells. The pathogens of TSEs might first colonize and then invade host cells, and only then would the infected cells (the focus) produce substances toxic to the target neurons, as proposed for the pathogenesis of other intracellular pathogen infections [5–7]. It is an important notion in the PHS hypothesis that substances derived from injured neurons affected by the initial insult could induce further inflammation, and a host with improper immune function against those substances would thus be expected to endure disease progression. In that scenario, amyloid proteins, including the products of the PrP gene, are primary immune effectors or supporting proteins for controlling etiological or toxic substances. For example, a host whose immune proteins are associated with the normal PrP gene can effectively control toxic substances, but a host with variant proteins from a modified PrP gene might be less able or unable to do so. Also, it could explain why the mice with absence of PrP gene reduce or eliminate the immune reaction against etiological substances [83]. It is possible that the products of PrP gene may be primary immune effectors, just like that T cell–deficient mice show less severe or no pulmonary lesions in viral or mycoplasma pneumonia models, suggested that T cells may be main effectors against the pathogenic peptides affecting target-respiratory cells and induce the peptides-associated inflammation [4, 5, 64, 65]. Also, it is possible that PrP ^Sc^, which is isoforms of normal gene products with changed conformational forms, are more effective effectors and have a suitable structure for binding to the etiological substance, thereby transmitting the disease.

Amyloidosis is a group of illnesses in which patients exhibit an abnormal accumulation of host protein or peptide molecules bound together in large, insoluble, protease-resistant aggregates [91]. Those aggregates can systemically or locally affect nearly any organ tissue, including cells in the kidneys, heart, lungs, gastrointestinal tract, soft tissues, and skin. They are usually fibrillar when visualized by electronic microscopy and can be detected by the Congo red stain method. The pathophysiology of amyloidosis remains unknown, but the associated diseases include those with genetic traits, such as ATTRv and AFib, and those with underlying diseases that are accompanied by abnormal immune reactions, such as AL and AA amyloidosis [91]. Furthermore, the clinical manifestations and pathologic findings, such as the components of the deposited amyloidogenic proteins and peptides, are different in each disease, whereas the structure and properties of the protein aggregates, such as their insolubility, protease resistance, birefringent staining with Congo red, and high beta-sheet content, are similar across diseases, as observed in the prion diseases. These findings suggest that a common immunopathogenesis is involved in prion diseases and non-prion amyloid diseases [92].

Certain chronic progressive CNS diseases, including AD, PD, and HD, are associated with genetic traits, similar to familial CJG and GSS in prion diseases. The pathological hallmarks of these diseases include intracellular inclusion bodies, extracellular amyloid deposits with various components, and neuronal loss. Well-studied amyloid proteins are amyloid beta (Aβ) and phosphorylated tau in AD and α-synuclein in PD. Inclusion bodies and extracellular amyloid proteins, such as Lewy bodies in PD and neurofibrillary tangles (tau protein) in AD, are considered to play important etiopathogenetic roles in neurodegenerative diseases. However, some affected patients have no intracellular inclusion lesions, and the degree of inclusion-body involvement in the pathology findings is not related to the clinical severity of the diseases. Furthermore, multiple risk factors are related to the diseases [93]. There are genetic forms of these diseases, and experimental studies of gene-null mice for the tau protein or α-synuclein show that those genes do not play a significant role in disease phenotype, pathologic lesions, or disease progression [94, 95]. Thus, the intracellular inclusion bodies and extracellular amyloid proteins might be associated with the immune reaction, similar to the findings for proteins of PrP gene in prion disease. Despite the similarity of pathological and clinical characteristics across neurodegenerative and prion diseases, attempts to unify the pathogenesis of these diseases has been controversial [96, 97].

According to the PHS hypothesis, a protein deficiency or a malfunctional protein within cells or the host can be corrected in part by the production of an alternative protein to replace the missing normal protein [6]. Furthermore, the long-term action of the alternative (nonspecific) protein can be associated with cellular injury in genetic diseases (see below). In neurodegenerative and amyloid diseases, various etiological substances target different neuronal cells, and the corresponding immune reaction differs in each disease. Also, alternative or defective proteins for immune-associated proteins, including proteins of PrP variant gene, can induce improper immune status against toxic substances. As result of those immune processes, corpses of soldiers are remained in the battle fields as amyloid aggregates with corresponding gliosis, damaged neurons, and possibly toxic enemies (etiological substances). One characteristic of immune reactions in neurodegenerative diseases is slow progression. That fits the PHS hypothesis because the incubation time and prognosis of a disease depends on the amount of etiological substance and corresponding immune status of the host, including the pool of immune proteins, which is genetically determined on an individual basis. Immune system functioning decreases in old age, and increasing age is the greatest risk factor for neurodegenerative diseases. Likewise, the proposed immune protein systems also fade with age.

## Genetic neurologic diseases

There are hundreds of genetic diseases of the CNS. The defective proteins in genetic disorders include structural proteins for neurotransmitter receptors and other receptors or ion channels on CNS cells, and proteins involved in enzymatic process, metabolism (transport), or signal transduction pathways in various communication systems [98]. Because a discussion of each genetic disease is beyond the scope of this review, only crucial points about the pathogenesis of genetic diseases are discussed. Single-gene defect diseases of the CNS can be caused by a defective product from a gene, i.e., a protein deficiency or a malfunctioning protein. In general, autosomal dominant genetic diseases are caused by structural protein defects, and autosomal recessive diseases are caused by defects in enzymatic proteins. However, certain genetic diseases that involve an enzymatic or multifunctional protein defect can induce structural cell injury during the natural course of the illness.

Patients with genetic diseases, including HD, familial JCD, GSS, and the genetic forms of AD and PD, show different clinical manifestations from other affected people in their family, including the time of onset of neurological symptoms, speed of progression of the disease, and prognosis, suggesting that phenotypes can vary even when the genotypes are identical. Likewise, similar phenotypes of CNS symptoms can be found in different genetic diseases. In genetic animal models, the phenotypes of single gene knockout can vary by strain in mice, and the clinical manifestations of a gene defect can differ between mice and humans, and mice null for some genes have also no observable phenotypic abnormalities compared with controls [99]. These findings suggest that default of a protein might be at least partly controlled by individual’s control systems and that there might exist a similar immune/repair system against cell injury in genetic diseases.

The pathophysiology of most genetic diseases in the CNS is complex because any affected gene is associated with numerous proteins and their corresponding activations of genes and epigenetic changes that occur during disease processes. Thus, the use of a genetic marker for diagnosing or predicting a prognosis remains impractical in clinical settings [100]. Susceptibility to immune-mediated diseases has been explained through epigenetic and environmental factors, which have improved understanding of the pathophysiology of disease. Studies of epigenetic mechanisms, which modulate gene expression without changing the genetic code, have suggested a link between genetic factors and acquired predisposing factors for disease [101]. Well-studied epigenetic factors in neurologic diseases include DNA methylation, histone modifications, and non-coding RNA, including micro-RNA and long non-coding RNA. Researchers have evaluated relationships among epigenetic factors in CNS diseases including AD and PD [102, 103]. An analysis of epigenetic factors could be useful for detecting diagnostic or prognostic biomarkers and developing targeted therapies for neurodegenerative diseases. On the other hand, epigenetic changes are observed not only in chronic neurodegenerative disorders but also in acute disorders, such as stroke and traumatic brain injury [104]. Also, it remains unknown whether the epigenetic alterations observed in various conditions contribute to disease development or are secondary or adaptive phenomena indicating immune reactions to insults caused by the disease.

The mechanisms of cell injury in genetic diseases are partially understood. The PHS hypothesis proposes that alternative or compensatory proteins produced in response to specific protein deficiencies can act in patients with genetic defects. Thus, patients who can produce mostly accurate or functional alternative proteins might have a less severe clinical course and better prognosis than those who cannot [6]. Also, limitations on the activity of defective proteins caused by a mutant gene or prolonged activation of alternative proteins might be associated with host cell injury caused by a failure to control toxic substances or easy vulnerability to cell injury from infectious or physical insults. Moreover, host cells injured by that mechanism could induce the sterile inflammation if those substances are released outside the affected cells [105]. The immune functions of the host, including the immune protein repertoire, control substances toxic to CNS cells. Thus, it is possible that continuous activation of nonspecific immune reactions caused by alternative or malfunctioning proteins, such as products of PrP variant genes and products of certain single nucleotide polymorphism or mutation of genes for immune regulation, could cause chronic inflammation in certain genetic diseases, as like that persistent activation of nonspecific adaptive immune systems may be responsible for autoimmune diseases.

Although there are few effective drugs for treating genetic diseases, immune modulators, especially corticosteroids, can effectively delay the progression of some genetic disorders, including Duchenne muscular dystrophy and other dystrophinopathies [106], as well as most autoimmune diseases. These findings suggest that there is a sterile inflammation in certain genetic diseases. It is possible that immune modulators, including corticosteroids, and possibly drugs for epigenetic regulation in immune reactions, suppress the immune reactions caused by nonspecific immune components, including alternative proteins for immune proteins, and thereby alleviate the symptoms and signs of disease.

## Conclusions

CNS diseases are heterogeneous entities with unresolved etiologies and pathophysiology. Researchers have explained the immunopathogenesis of many diseases, including CNS diseases, using an established immunological dogma in which T cells are the central player in immune regulation. However, that dogma has some limitations in solving the puzzles of disease pathophysiology. Although the immune system is divided into two major subsystems, all immune cells and immune proteins in both systems perform their functions during noninfectious events, such as trauma (or wound healing), transplantation rejection, intoxication, and cancers. Also, the components of the host immune system control not only pathogens but also smaller substances produced by infectious agents or host cells injured by infectious insults. Thus, all biological responses observed during a disease insult could be controlled by an integrated and translational control system of the host. The PHS hypothesis suggests that every disease involves an etiological substance, and the main function of the host immune system is to control toxic substances and protect cells according to the size and biochemical properties of the substances. Thus, the phenotype of a disease is determined by the etiological or inflammation-inducing substances, the target cells of those substances in organs, and the corresponding immune reaction to those substances. Also, the PHS hypothesis has provided the rationale for early use of immune modulators (corticosteroids and/or IVIG) for patients with acute infectious diseases, including influenza, *Mycoplasma pneumoniae* pneumonia, and COVID-19, and acute infection-related immune-mediated diseases including KD, MIS-C, and other acute organ-specific diseases [7, 64, 65, 67, 107, 108].

This article proposed a common immunopathogenesis of CNS diseases, including prion diseases, chronic neurodegenerative diseases, and genetic diseases, through the PHS hypothesis. I believe that the etiological substances, the focus of them, the target cells, and corresponding immune components in each CNS disease would be defined in the future, and proper diagnosing, treatment, and prevention modalities for the diseases will be developed. I hope that this article helps researchers solve the remaining puzzles about the etiology, pathophysiology, and treatment of CNS diseases.

## Data Availability

Not applicable.

## Abbreviations

ADEM: Acute disseminated encephalomyelitis
AD: Alzheimer disease
BBB: Blood–brain barrier
BSE: Bovine spongiform encephalopathy
CSF: Cerebrospinal fluid
CNS: Central nervous system
CWD: Chronic wasting disease
CJD: Creutzfeldt-Jakob disease
DAMPs: Damage (danger)-associated molecular patterns
FFI: Fatal familial insomnia
GSS: Gerstmann-Sträussler-Scheinker syndrome
GBS: Guillain–Barre syndrome
HD: Huntington’s disease
IL: Interleukin: IVIG: Intravenous immunoglobulin
JIA: Juvenile idiopathic arthritis
KD: Kawasaki disease
MHC: Major histocompatibility complex
MCs: Mast cells
MCAS: Mast cell activation syndrome
MIS-C: Multisystem inflammatory syndrome in children
PD: Parkinson’s disease
PAMPs: Pathogen-associated molecular patterns
PRRs: Pattern recognizing receptors
PHS: Protein-homeostasis-system
SLE: Systemic lupus erythematosus
TCR: T cell receptor
TLRs: Toll-like receptors
TSEs: Transmissible spongiform encephalopathies
TNF-α: Tumor necrosis factor-α
UPS: Ubiquitin–proteasome system
